# Correlation between Retinal Changes and Visual Function in Late-Stage Vogt-Koyanagi-Harada Disease: An Optical Coherence Tomography Study

**DOI:** 10.1155/2015/916485

**Published:** 2015-08-02

**Authors:** Min Zhou, Chunhui Jiang, Ruiping Gu, Zhongcui Sun, Nancy Huynh, Qing Chang

**Affiliations:** ^1^Department of Ophthalmology, Eye and ENT Hospital, Fudan University, Fenyang Road 83, Xuhui District, Shanghai 200031, China; ^2^Shanghai Key Laboratory of Visual Impairment and Restoration, Fudan University, China; ^3^The Southeast Permanente Medical Group, Atlanta, GA, USA

## Abstract

*Purpose.* To characterize the optical coherence tomography (OCT) findings in late-stage Vogt-Koyanagi-Harada (VKH) disease and its correlation with visual function.* Methods.* The records of patients with late-stage VKH disease (defined as ≥12 months from disease onset) were retrospectively reviewed. The analysis focused on the OCT findings and microperimetry, in addition to the possible correlation between morphology and functional findings.* Results.* Twenty-nine patients (58 eyes) were included. Mean age at onset was 34.24 ± 10.67 years. The OCT revealed that the outer retina and retinal pigment epithelium (RPE) were mainly affected. These effects included RPE thickening and breakage or disappearance of the cone outer segment tip (COST) line and/or inner segment/outer segment (IS/OS) junction. The COST line and IS/OS results were related to macular function and the interval between symptom onset and initiation of high-dose corticosteroid treatment (all *P* < 0.01). Eyes with intact COST lines demonstrated intact IS/OS and normal RPE layers as well as better visual function and normal retinal sensitivity.* Conclusions.* The OCT findings are strongly correlated with macular function, as well as other clinical findings in late-stage VKH. With respect to the COST line and retinal sensitivity especially, the OCT and microperimetry findings may be useful for evaluating later-stage VKH.

## 1. Introduction

Vogt-Koyanagi-Harada disease (VKH) is a granulomatous inflammatory disorder affecting the eyes, auditory system, meninges, and skin [1, 2]. It is one of the most common causes of uveitis in Asia and accounts for approximately 16% of all uveitis cases in China [3]. Clinically, VKH is divided into four stages: prodromal, acute, convalescent, and chronic recurrent. Exudative retinal detachment is a common cause of visual function impairment among VKH patients [4], but secondary alterations in retinal morphology and function also occur [5, 6]. Due to advancements in retinal imaging, such as fundus autofluorescence (FAF) [7, 8] and optical coherence tomography (OCT) [9, 10], important clinical insight into the pathophysiology of the retina can be achieved noninvasively. Recent studies report a significant association of the inner segment (IS)/OS junction and the cone outer segment tips (COST) line in various retinal diseases with visual function [11–13]. However, the majority of previous studies have used best corrected visual acuity (BCVA) as a measure of visual function. Recently, microperimetry was found to provide a more comprehensive assessment of macular sensitivity and functional changes in the macula [14, 15].

We investigated the characteristics of late-stage VKH via OCT, especially photoreceptor microstructure integrity and its potential correlation to visual function.

## 2. Methods

### 2.1. Study Participants

Patients with late-stage VKH (defined as ≥12 months from disease onset) who visited the uveitis clinic at Fudan University Eye and ENT Hospital (Shanghai, China) between April 2011 and March 2013 were enrolled in this cross-sectional study. The diagnosis of VKH was made according to the Revised Diagnostic Criteria proposed by the International Nomenclature Committee [2]. The inclusion criteria were treatment with high-dose corticosteroid therapy (IV methylprednisolone at 1 g/d for 3 d) followed by a slow tapering of the drug dose over a 6-month period, undergoing a thorough examination, including OCT and microperimetry, and providing the required information at the 12-month follow-up, including sex, age at VKH onset, BCVA at initial presentation, current BCVA, interval from the onset of VKH symptoms to the initiation of high-dose corticosteroid treatment, and current ocular inflammatory status. The clinical characteristics were classified according to the Standardization of Uveitis Nomenclature Working Group [16]. Exclusion criteria were inadequate mydriasis (<7 mm), media opacities (caused by cataract or other), and glaucoma (defined as characteristic optic nerve damage or typical visual field defects accompanied by compatible optic nerve damage). This study was conducted in accordance with the tenets of the Declaration of Helsinki and was approved by the local ethics committee of the Fudan University Eye and ENT Hospital.

### 2.2. SD-OCT

OCT images of dilated pupils were captured via SPECTRALIS SD-OCT (ver. 1.5.12.0, Heidelberg Engineering, Heidelberg, Germany) using the volume scan mode (1024 × 25; 25 lines, 1024 A-scans per line, each line comprising 30 averaged scans obtained using eye tracking), which covered a 30 × 30 degree area centered on the fovea. Each line was assigned a unique serial number. Microperimetry covered the central 12° of the macula; therefore, only nine scans at the central macula (firstly the scan across the central fovea was found and then subsequent four scans in both the superior and inferior directions were picked), which covers about 12° of the central macula, were selected for further analysis. Changes in the IS/OS junction, the COST line, and the RPE layer were graded by an experienced ophthalmologist (Chunhui Jiang), who was masked for the clinical and other findings of the patients. Grading was performed using a scale from 1 to 3 based on a standardized protocol (Table 1). Also if one part of the outer nuclear layer (ONL) was thinner than the other, or all ONL was obviously much thinner than normal, then it was considered as thinning of ONL.

### 2.3. Microperimetry

All patients underwent microperimetry under cycloplegia using the MP-1 microperimeter (software ver. SW1.4.1 SP1; Nidek Technologies, Padua, Italy) [17]. Briefly, Goldmann III target and a 4–2 staircase strategy were used. Forty-five stimulus locations covering an area of 12° in diameter were examined. Based on a 4–2 staircase strategy on a set of 45 Goldmann III stimulating spots covering the central 12° of the fundus, all tests were performed using an automated pattern in which a 1° circle was the target. The intensity of each stimulus was varied according to a 1-step (0.1−log) scale from 0–20 decibels (dB) for an equivalent duration of 200 ms per step. The software automatically calculated the mean sensitivity, which was defined as the average of all 45 scores.

### 2.4. Statistical Analysis

All analyses were performed using a statistical software package (SPSS for Windows, ver. 17.0; SPSS, Inc., Chicago, IL, USA). Differences in MP and BCVA between groups of late-stage VKH eyes with different OCT grading were examined using the mixed linear model test. Differences in each prognostic factor (sex, age of onset, initial BCVA, and interval from onset to treatment) were analyzed using Generalized Estimation Equations test. The durations between patients who were still using systemic corticosteroids alone or in combination with an immunosuppressant or not at 12 months were analyzed using Mann-Whitney *U* test. A *P* value of <0.05 was considered statistically significant.

## 3. Results

### 3.1. Patient Characteristics

During the study period, 34 VKH patients with a follow-up of longer than 12 months visited our clinic. Twenty-nine patients (15 males and 14 females, 58 eyes) who met the inclusion criteria were enrolled in the study. The mean follow-up time was 12.2 months (range 12–14 months). The mean age at onset was 34.24 ± 10.67 years (range 12–65 years); mean BCVA at onset was 0.96 ± 0.56 (logMAR, range 2.00–0); mean BCVA at 12 months was 0.07 ± 0.16 (logMAR, range 0.40 to −0.08 logMAR); and mean retinal sensitivity was 17.05 ± 2.35 dB (range 9.9–20 dB). At 12 months, 16 of the 29 patients were still using systemic corticosteroids alone or in combination with an immunosuppressant. Among them, 10 patients were tapering down and using low-dose prednisone. Another six patients were receiving corticosteroids and cyclosporine prescribed at 3 mg/kg/d and were tapering, guided by the severity of ocular inflammation.

### 3.2. SD-OCT Findings

**Figure 1:**
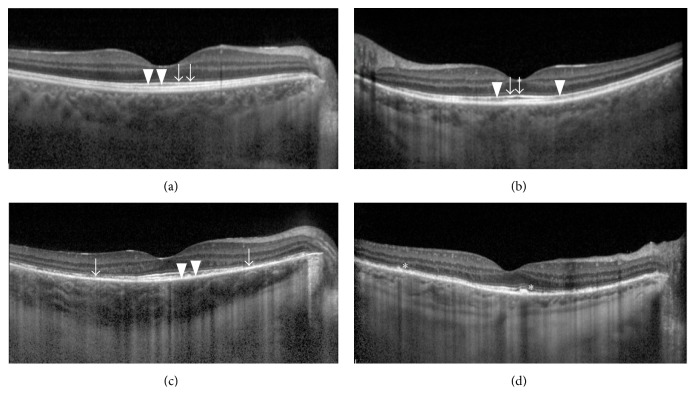
Standards for OCT grading: (a) intact COST line (arrowheads) and junction between the photoreceptor inner and outer segments (IS/OS) line (arrows) with normal RPE layer; (b) discontinuous COST lines (arrowheads) and intact IS/OS line (arrows); (c) discontinuous COST line (arrowheads) and IS/OS line (arrows); and (d) thickened RPE layer (asterisk).

The OCT findings in the late-stage VKH eyes were localized to the outer retina. The findings included thickening of the RPE layer (28/58 eyes); breakage or disappearance of the COST line (51/58 eyes) and/or the inner segment/outer segment (IS/OS) junction (28/58 eyes); and thinning of the outer nuclear layer (ONL) (17/58 eyes). The grading of the RPE, COST line, and IS/OS conjunction is listed in Table 2. Notably, all eyes displaying intact COST lines also displayed intact IS/OS junctions and normal RPE layers. However, 23 of the 30 eyes displaying intact IS/OS junctions displayed fragmented (Grade 1, 3 eyes; Grade 2, 20 eyes) COST lines. In 18 of the 51 eyes displaying fragmented COST lines (Grade 1 or 2) and in 16 of the 28 eyes displaying fragmented IS/OS junctions (Grade 1 or 2), the central fovea remained intact (Figures 1(a)–1(c)).

### 3.3. Correlation between the SD-OCT Findings and Visual Function

**Figure 2:**
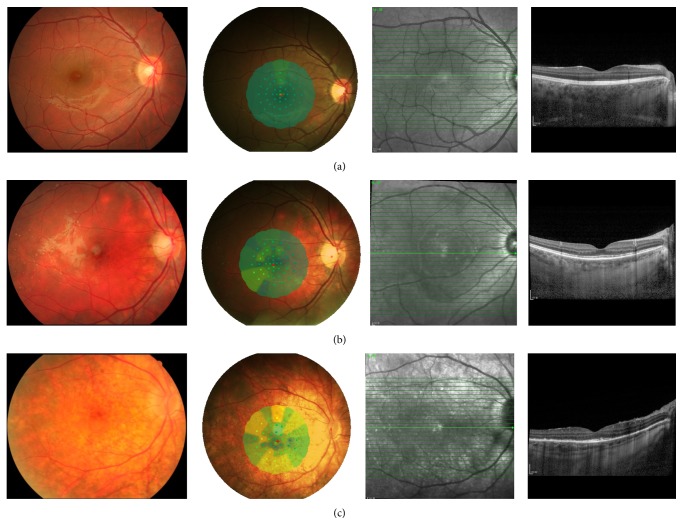
Fundus appearance, microperimetry, and OCT findings in different groups (12 months after disease onset). (a) (COST+/IS/OS+): a rather normal fundus; mean retinal sensitivity 19.50 dB; OCT image demonstrated intact COST and junction between the photoreceptor inner and outer segment (IS/OS) lines. (b) (COST−/IS/OS+): a “sunset glow” fundus; mean retinal sensitivity 18.00 dB; OCT shows fragmented COST line and intact IS/OS line. (c) (COST−/IS/OS−): severe sunset glow fundus with hyperpigmentation; mean retinal sensitivity 15.80 dB; OCT demonstrated absent COST line and fragmented IS/OS line with thickened RPE layer.

Our analysis revealed a strong correlation between retinal sensitivity/BCVA and the OCT findings of the COST line, IS/OS junction, and RPE layer (all *P* ≤ 0.01, Table 2) (Figure 2). For clarification, the patients were categorized according to the COST line and IS/OS junction characteristics, which were graded as intact (+) or not (−) (Table 3). The three groups had similar gender/age distributions (Table 3) but different BCVA and retinal sensitivities. The IS/OS+/COST+ group displayed the best retinal sensitivity and BCVA; furthermore, only this group displayed relatively normal retinal sensitivity (Table 3).

### 3.4. Correlation between the SD-OCT Findings and the Clinical Characteristics of the Patients

The OCT findings for the COST line and IS/OS junction displayed clear correlations to the interval from symptom onset to the initiation of high-dose corticosteroid treatment and usage of systemic corticosteroids and/or immunosuppressant at 12 months after onset (Table 3). The IS/OS+/COST+ group exhibited the smallest interval from symptom onset to treatment and no usage of systemic corticosteroids and/or immunosuppressant. In contrast, 11/23 and 21/28 patients in the IS/OS+/COST− and IS/OS−/COST− groups were using systemic corticosteroids alone or in combination with an immunosuppressant. Furthermore, all patients receiving immunosuppressant treatment were in the IS/OS−/COST− group. Patients who were using systemic corticosteroids alone or in combination with an immunosuppressant at 12 months had longer durations than those without (using versus not using, median 30 versus 9, *P* < 0.0001 by the Mann-Whitney *U* test).

## 4. Discussion

Changes of the outer retina and RPE in late-stage VKH patients, including thickening of the RPE layer, disruption or loss of the COST line and IS/OS junction, and thinning of the ONL, were clearly demonstrated by SD-OCT. Further analysis revealed a close correlation between these OCT findings, which also correlated to the BCVA, retinal sensitivity, and clinical characteristics. Better OCT findings correlated to better vision, prompt treatment at disease onset, and less incidence of systemic corticosteroids and/or immunosuppressant at 12 months after onset.

Clinical findings of late VKH patients vary greatly. da Silva et al. recently reported that, in late-stage VKH eyes (>6 months past disease onset), grading by color fundus photos (which was mainly based on diffuse fundus depigmentation or subretinal fibrosis) correlated well with full-field electroretinogram (ERG) results [5]. However, not all VKH patients had diffuse fundus depigmentation or subretinal fibrosis; therefore, an objective method with higher resolution might be useful in the study of VKH patients with less fundus change. OCT is routinely used in the diagnosis and follow-up of many retinal diseases [9–13]. Ishihara et al. recently reported an OCT study of acute VKH patients [4], and Koizumi et al. reported defects in the IS/OS junction and multifocal thickening of the RPE in late-stage VKH patients [8]. However, the use of OCT to identify retinal morphological changes in late-stage VKH patients has not been extensively studied [5, 7, 8, 18]. This study comprised a relatively large series of late-stage VKH patients, in whom OCT clearly demonstrated retinal changes and RPE. In addition to defects in the IS/OS junction and thickening of the RPE, thinning of ONL and a high prevalence (51/58) of defects in the COST line were found, thereby indicating that OCT is useful in the observation of late-stage VKH eyes. All eyes displaying intact COST lines displayed normal IS/OS junctions or RPE layers, whereas those displaying intact IS/OS junctions or normal RPE layers displayed disrupted COST lines. It has been reported in central serous chorioretinopathy [11] or macular hole [12] patients that the COST line is later to recover than are the IS/OS line and other characteristics. Notably, patients displaying COST line and IS/OS junction defects or loss at the perifovea exhibited intact structures at the central fovea. The mechanism underlying this finding is not fully understood, but the high density of photoreceptors at the fovea may play an important role [19]. Alternatively, the cones at the fovea display a different configuration and arrangement than cones at other regions of the fundus [19], which might also explain these findings. Whether these structures were preserved or were first to regenerate, studying the mechanism behind this characteristic might improve our understanding of VKH and of the neuroprotection or neuroregeneration of the retina.

Our study also demonstrated a strong correlation between the COST line and/or IS/OS junction integrity and visual function in late-stage VKH patients. A former paper reported a similar correlation in patients with macular holes [12, 20]. However, BCVA was employed as the measure of visual function in previous studies. This time, microperimetry, which provides a quantitative measurement of retinal sensitivity at the macula, was also used. Using microperimetry, we found that only the IS/OS+/COST+ eyes exhibited normal results (IS/OS+/COST+ group: 19.56 ± 0.36 dB; normal group: 19.1 ± 0.9 dB) [13]. These results suggest that the presence or reappearance of an intact COST line might serve as a more sensitive indicator of photoreceptor functional status than that of the IS/OS junction. In accordance with Yokota's report of a higher cone density in eyes displaying intact COST lines than in eyes displaying disrupted COST lines [21], our finding supported Fujita and Itoh's theory that the recovery of the COST line, but not the IS/OS junction, suggests enhanced restoration of the photoreceptors [11, 12]. On the other hand, it might also suggest that retinal sensitivity measured by microperimeter appeared to be able to provide a comprehensive and accurate evaluation of macular function in late-stage VKH patients and might do the same in patients with other retinal diseases.

The OCT findings were also related to the mean interval from symptom onset to the initiation of systemic steroid therapy and using of systemic corticosteroids and/or immunosuppressant at 12 months after onset (Table 3). Therefore, the interval from symptom onset to the initiation of systemic steroid therapy is an important prognostic factor for patients with late-stage VKH. Thus, proper and prompt treatment is crucial for VKH patients. Chee reported that early treatment (<2 weeks) of high-dose corticosteroids was associated with better visual results in VKH patients [6]. However, the study used BCVA as an indicator. Our IS/OS+/COST− group displayed a relatively normal BCVA and an average treatment interval of 20.22 ± 13.12 days. However, this group did not exhibit normal retinal sensitivity. In contrast, only the COST+/IS/OS+ group exhibited normal retinal sensitivity, normal BCVA, and treatment interval of 6.71 ± 0.76 days. This result suggests that more prompt treatment might be required to achieve complete restoration of macular structure and function. The importance of prompt treatment was also suggested by the correlation with less incidence of systemic corticosteroids and/or immunosuppressant at 12 months after onset.

The strengths of our study include its well-defined population who underwent standard long-term follow-up. A relatively large sample size was achieved by using sophisticated statistics like “mixed linear model” and others. These methods correct the dependence of both eyes from the same patients, so both eyes of the patients could be included. The primary outcome measures used in this study (OCT findings, microperimetry, retinal sensitivity, and BCVA) are largely objective measures that are easily quantifiable. The study's retrospective design is a limitation, as is the fact that longitudinal assessment of disease activity and visual function could not be assessed due to the use of a cross-sectional study design. The mechanisms underlying particular findings, such as the preservation of the retinal structure at the fovea, are not fully understood. Further follow-up study might be able to tell us more about how these VKH patients recovered from their acute episode and how the functional and anatomic recovery were correlated.

In summary, our study demonstrated a significant correlation between the OCT findings and visual function in late-stage VKH patients, as determined via BCVA and microperimetry. The integrity of the COST line was more sensitive than that of the IS/OS junction. Moreover, prompt initiation of high-dose corticosteroid treatment was associated with improved prognosis for visual function, retinal structure, and less incidence of systemic corticosteroids and/or immunosuppressant at 12 months.

## Figures and Tables

**Table 1:** Grading protocol for the COST line, IS/OS junction, and RPE layer thickness.

	Grade 1	Grade 2	Grade 3
COST line	Continuous and intact on all nine scans	Discontinuous on less than half (<5) of the nine scans	Discontinuous on more than half (≥5) of the nine scans

IS/OS junction	Continuous and intact on all nine scans	Discontinuous on less than half (<5) of the nine scans	Discontinuous on more than half (≥5) of the nine scans

RPE layer	Clear and smooth RPE layer on all nine scans	Thickened RPE layer on less than half (<5) of the nine scans	Thickened RPE layer on more than half (≥5) of the nine scans

COST line; IS/OS: junction between the photoreceptor inner and outer segments.

RPE: retinal pigment epithelium.

**Table 2:** Visual function of 58 late-stage (12 months after disease onset) VKH eyes with different OCT gradings.

	Grade	1	2	3	Total
COST line^*∗∗*^	Number	7	3	48	58
	Percentage	12.07%	5.17%	82.76%	100%
	BCVA (logMAR)	−0.05 ± 0.04	0.0 ± 0.00	0.08 ± 0.11	
	Retinal sensitivity	19.56 ± 0.36	18.63 ± 1.12	15.58 ± 2.30	

IS/OS junction^*∗∗*^	Number	30	9	19	58
	Percentage	51.72%	15.52%	32.76%	100%
	BCVA (logMAR)	0.016 ± 0.058	0.0356 ± 0.072	0.1453 ± 0.142	
	Retinal sensitivity	18.30 ± 1.53	16.86 ± 2.18	15.30 ± 2.36	

RPE layer^*∗∗*^	Number	30	12	16	58
	Percentage	51.72%	20.69%	27.59%	100%
	BCVA (logMAR)	0.013 ± 0.064	0.517 ± 0.065	0.159 ± 0.146	
	Retinal sensitivity	18.15 ± 1.52	16.98 ± 2.26	15.03 ± 2.44	

COST line; IS/OS: junction between the photoreceptor inner and outer segments.

RPE: retinal pigment epithelium.

BCVA: best corrected visual activity.

Grade 1: continuous and intact on all scans; Grade 2: discontinuous on less than half (<5) of the nine scans; Grade 3: discontinuous on more than half (≥5) of the nine scans.

^*∗∗*^ *P* ≤ 0.01 among all three groups based on a mixed linear model test for both MP and BCVA.

**Table 3:** Visual function and other clinical findings in 29 late-stage VKH patients with different IS/OS and COST findings.

Patient characteristics	OCT grading			*P* value
	COST+ IS/OS+ Group 1	COST− IS/OS+ Group 2	COST− IS/OS− Group 3	All compared
Number of eyes	7	23	28	
Age	32.14 ± 3.58	36.61 ± 11.20	32.82 ± 11.29	0.394^*∗*^
Gender (male)	57.14%	52.17%	50.00%	0.803^*∗*^
Retinal sensitivity (dB)	19.56 ± 0.36	17.92 ± 1.54	15.715 ± 2.35	<0.001
BCVA (logMAR)	−0.05 ± 0.04	0.03 ± 0.05	0.11 ± 0.13	0.001
Incidence of systemic corticosteroids and/or immunosuppressant at 12 months	0 (0%)	11 (18.97%)	21 (36.21%)	<0.0001
Interval between onset and treatment (days, mean ± SD)	6.71 ± 0.76	20.22 ± 13.12	33.79 ± 17.49	<0.001

OCT means optical coherence tomography; BCVA means best correct visual activity. Group 1: COST lines and IS/OS lines were all continuous and intact on all scans; Group 2: IS/OS lines were continuous and intact on all scans, but COST lines were discontinuous on one or more scans; Group 3: either COST lines or IS/OS lines were discontinuous on one or more scans. The Generalized Estimation Equations test was used to compare differences between all three groups.

^*∗*^Not statistically significant.
